# The Mature COC Promotes the Ampullary NPPC Required for Sperm Release from Porcine Oviduct Cells

**DOI:** 10.3390/ijms24043118

**Published:** 2023-02-04

**Authors:** Zhanying Wu, Biao Li, Kaiwei Yu, Nana Zheng, Feifei Yuan, Jingjing Miao, Meijia Zhang, Zhijuan Wang

**Affiliations:** 1 Division of Cell, Developmental and Integrative Biology, School of Medicine, South China University of Technology, Guangzhou 510006, China; 2 State Key Laboratory for Agrobiotechnology, College of Biological Sciences, China Agricultural University, Beijing 100193, China

**Keywords:** NPPC, CNG channel, TGF-β signaling, sperm release, oviduct

## Abstract

Porcine spermatozoa are stored in the oviductal isthmus after natural mating, and the number of spermatozoa is increased in the oviductal ampulla when the mature cumulus-oocyte complexes (COCs) are transferred into the ampulla. However, the mechanism is unclear. Herein, natriuretic peptide type C (NPPC) was mainly expressed in porcine ampullary epithelial cells, whereas its cognate receptor natriuretic peptide receptor 2 (NPR2) was located on the neck and the midpiece of porcine spermatozoa. NPPC increased sperm motility and intracellular Ca^2+^ levels, and induced sperm release from oviduct isthmic cell aggregates. These actions of NPPC were blocked by the cyclic guanosine monophosphate (cGMP)-sensitive cyclic nucleotide-gated (CNG) channel inhibitor *l-cis*-Diltiazem. Moreover, porcine COCs acquired the ability to promote NPPC expression in the ampullary epithelial cells when the immature COCs were induced to maturation by epidermal growth factor (EGF). Simultaneously, transforming growth factor-β ligand 1 (TGFB1) levels were dramatically increased in the cumulus cells of the mature COCs. The addition of TGFB1 promoted NPPC expression in the ampullary epithelial cells, and the mature COC-induced NPPC was blocked by the transforming growth factor-β type 1 receptor (TGFBR1) inhibitor SD208. Taken together, the mature COCs promote NPPC expression in the ampullae via TGF-β signaling, and NPPC is required for the release of porcine spermatozoa from the oviduct isthmic cells.

## 1. Introduction

In many mammalian species, a subpopulation of the ejaculated spermatozoa migrates to the oviduct isthmus to form the “functional sperm reservoir”. The spermatozoa are held in the isthmic reservoir from a few hours to several months, depending on species [1], resulting in regulation of sperm functions, such as the low intracellular calcium concentration and the reduction of the capacitation-related protein tyrosine phosphorylation levels [2,3,4,5]. Near the time of ovulation, spermatozoa are released from the isthmic reservoir and migrate toward the site of fertilization in the oviduct ampulla where the mature cumulus-oocyte complexes (COCs) reside [6]. The sperm release from the isthmic reservoir is a prerequisite for fertilization. Several factors have been proposed to explain sperm release in the oviduct, such as oviduct peristaltic contractions, adovarian oviduct fluid flow [7] and oviduct fluid components (i.e., heparin and progesterone) [8,9].

During oestrus, luteinizing hormone (LH) from the pituitary acts on mural granulosa cells to induce the expression of epidermal growth factor (EGF)-like growth factors. Then, EGF receptor (EGFR) signaling is activated in cumulus cells, resulting in the maturation of COCs [10,11]. The mature COCs are picked up and transported into the oviductal ampulla for fertilization [12]. In pig, a significantly higher proportion of spermatozoa is found in the ampulla when the mature COCs are transferred into the oviductal ampulla [13]. In cow, spermatozoa bound in the reservoir are released from the isthmic reservoir as soon as the mature COCs arrive at the ampulla [14]. All these studies suggest that the interaction between COCs and ampulla is crucial for sperm release from the isthmic reservoir. Nevertheless, the molecular mechanism of sperm release from the isthmic reservoir is poorly understood.

It is reported that the mature COCs can alter mRNA and protein expression pattern in bovine and porcine oviduct epithelial cells, respectively [15,16]. Our previous study in mouse indicates that the mature COCs promote the expression of natriuretic peptide type C (NPPC) in the ampullae [17]. NPPC elevates intracellular Ca^2+^ levels of spermatozoa via cyclic guanosine monophosphate (cGMP)-sensitive cyclic nucleotide-gated (CNG) channels, and increases mouse and human sperm motility [17,18]. More importantly, spermatozoa derived from *Npr2* mutant mice are unable to reach the ampullae for fertilization [17]. Thus, the mature COC-induced NPPC in the ampullae may promote sperm release from the isthmic reservoir for fertilization by increasing sperm motility.

NPPC can be upregulated by transforming growth factor-β (TGFB) [19], estrogen [20], dexamethasone [21], and gonadotropin releasing hormone [22] in various cell types. Our previous study indicates that TGFB promotes NPPC expression in mouse granulosa cells to maintain oocyte meiotic arrest [23]. In mammals, the TGF-β family consists of three ligands, including TGFB1, TGFB2, and TGFB3 [24]. These ligands initiate signaling by binding to type 2 receptor (TGFBR2), which in turn recruits type 1 receptor (TGFBR1). Subsequently, receptor-associated SMAD (Sma- and Mad-related) proteins (R-SMADs), such as SMAD2 and SMAD3, are phosphorylated and form a heteromeric complex with SMAD4. Ultimately, the complex is translocated into the nucleus to activate transcription of the target genes [25]. TGF-β ligands are present in mouse cumulus cells of COCs [23,26], and their receptors are detected in mouse and human oviducts [27,28]. Thus, this work is designed to investigate the mechanism of NPPC expression in porcine ampullae and the function of NPPC in sperm release from the oviduct isthmic cells.

## 2. Results

### 2.1. Expression Pattern of NPPC in the Oviduct

**Figure 1:**
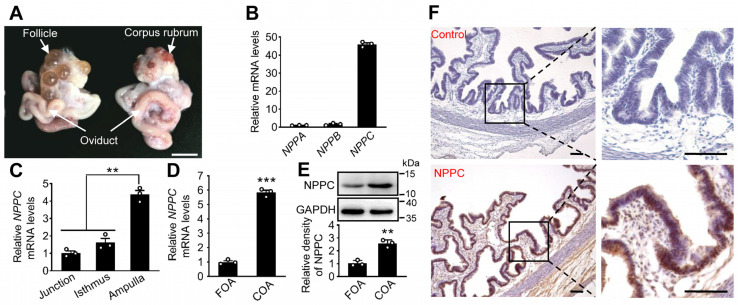
The expression pattern of NPPC in porcine oviduct. (**A**) Porcine oviducts at the follicular and corpus rubrum phase. Scale bar, 1 cm. (**B**) The mRNA levels of *NPPA*, *NPPB,* and *NPPC* in the ampullae at the corpus rubrum phase (*n* = 3 independent experiments). (**C**) The mRNA levels of *NPPC* in the uterotubal junction (junction), isthmus, and ampulla at the corpus rubrum phase (*n* = three independent experiments). (**D**,**E**) The mRNA (**D**) and protein (**E**) levels of NPPC in the ampullae at the follicular and corpus rubrum phase (*n* = 3 independent experiments). GAPDH was used as an internal control. (**F**) Immunohistochemistry showing NPPC expression in the ampulla. Scale bars, 100 μm. Representative examples of three independent experiments were shown. Bars indicate the mean ± SEM. Statistical analysis was performed by two-tailed unpaired Student’s *t*-test. ** *p* < 0.01 and *** *p* < 0.001. Each data point represents a biologically independent experiment. FOA, oviductal ampullae at follicular phase; COA, oviductal ampullae at corpus rubrum phase.

Firstly, we compared the mRNA abundance of natriuretic peptide ligands (NPPA, NPPB, and NPPC) in porcine ampullae from oviducts at the corpus rubrum phase (Figure 1A) using qRT-PCR. The relative abundance of *NPPC* mRNA was higher than that of *NPPB* and *NPPA* mRNAs (Figure 1B). We further compared the gene expression of NPPA, NPPB, or NPPC in the porcine oviductal ampulla and heart (the positive tissue). The results showed that the mRNA levels of *NPPA* and *NPPB* in the ampullae were significantly lower than those in the heart (Figure S1A). In contrast, the mRNA levels of *NPPC* in the ampulla were significantly higher than those in the heart (Figure S1A). These results suggest that NPPC is the predominant natriuretic peptide ligand in the ampullae. Next, we compared the levels of *NPPC* mRNA in the ampulla, isthmus, and uterotubal junction from the oviduct at the corpus rubrum phase. The results showed that the levels of *NPPC* mRNA in the ampulla were significantly higher than those in the uterotubal junction and isthmus (Figure 1C). Furthermore, the mRNA and protein levels of NPPC were significantly higher in the ampullae at corpus rubrum phase than those in the ampullae at follicular phase (Figure 1D,E). Immunohistochemistry results showed that NPPC was predominantly expressed in the ampullary epithelial cells (Figure 1F). Taken together, NPPC is mainly expressed in the ampullae at corpus rubrum phase.

### 2.2. Expression Pattern of NPR2 in Spermatozoa

**Figure 2:**
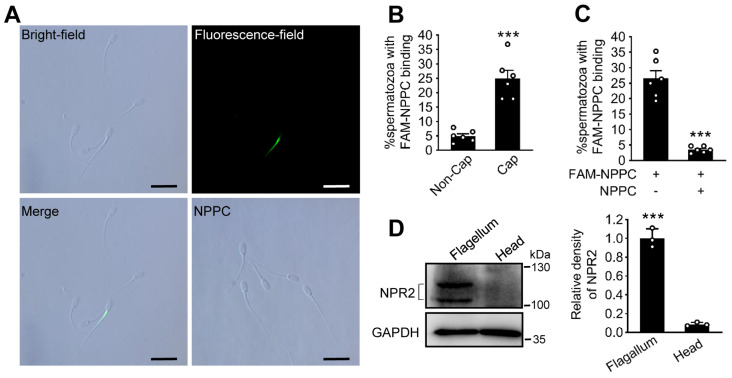
Expression of NPR2 on porcine sperm. (**A**) The capacitated spermatozoa were incubated with 100 nM FAM-NPPC for 30 min to ensure full reaction. The binding of FAM-NPPC to the spermatozoa was visualized by fluorescence and merge fields. Scale bars, 20 μm. Representative examples of three independent experiments are shown. (**B**) The percentage of spermatozoa with FAM-NPPC binding before and after capacitation (*n* = 6 independent experiments). (**C**) Comparison of FAM-NPPC binding in capacitated spermatozoa without or with 1 μM NPPC incubation (*n* = 6 independent experiments). (**D**) The protein levels of NPR2 in the capacitated sperm head and flagellum (*n* = 3 independent experiments). Two immunoreactive bands of NPR2 antibody were observed in the fraction of sperm flagellum. GAPDH was used as an internal control. Bars indicate mean ± SEM. Statistical analysis was performed by two-tailed unpaired Student’s *t*-test. *** *p* < 0.001. Each data point represents a biologically independent experiment. Non-Cap, non-capacitated spermatozoa; Cap, capacitated spermatozoa.

NPPC acts as an autocrine and paracrine regulator through NPR2, a guanylyl cyclase-coupled receptor [29]. *NPR2* mRNA could be detected in porcine spermatozoa, whereas *NPPC* mRNA was hardly detected (Figure S1B). To test the location of NPR2 on porcine spermatozoa, we used a fluorescent-labeled ligand NPPC (FAM-NPPC) to bind with the receptor. This method has been used to test the location of NPR2 in mouse spermatozoa in our previous study [17]. The green fluorescence, representing FAM-NPPC binding site, was observed on the neck and the midpiece of spermatozoa (Figure 2A). The positive signal was seen in 24.9 ± 2.9% of capacitated spermatozoa (Figure 2B). Few spermatozoa (3.5 ± 0.4%) showed fluorescence staining in the samples with competitive binding in the presence of excess unlabeled peptide (1 μM NPPC) (Figure 2C). These results indicate that NPR2 is located on the neck and the midpiece of porcine spermatozoa, the binding site of FAM-NPPC. However, the percentage of spermatozoa with FAM-NPPC-positive signal in non-capacitated spermatozoa was only 4.9 ± 0.8% (Figure 2B). We further used Western blotting to detect the endogenous levels of NPR2. The results showed that NPR2 was expressed predominantly in sperm flagellum (Figure 2D), consistent with our previous study in mouse [17]. Two immunoreactive bands (~130 kDa and ~117 kDa) were observed for NPR2 with different amounts of glycosylation.

### 2.3. NPPC Increases Sperm Motility and Intracellular cGMP and Ca^2+^ Levels

**Figure 3:**
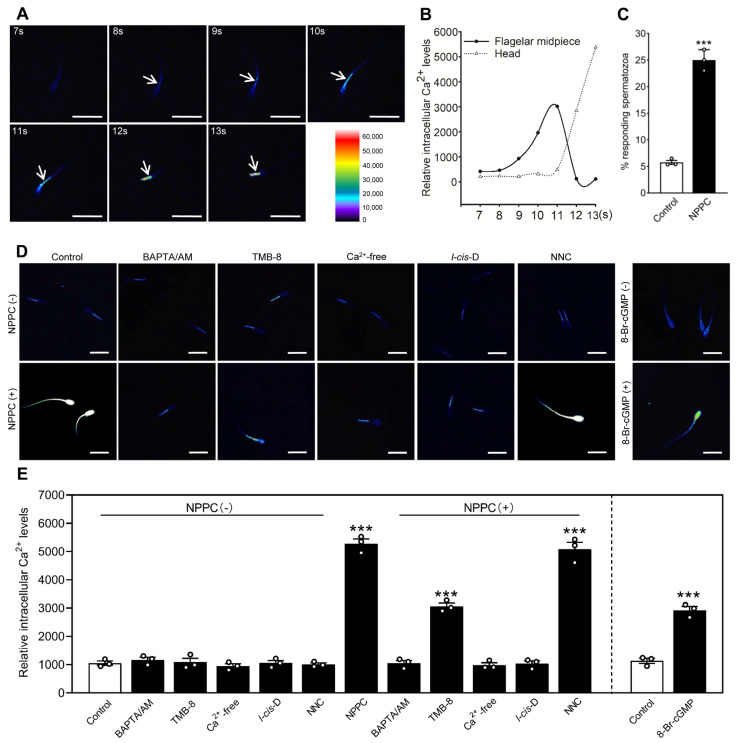
NPPC stimulates Ca^2+^ influx via CNG channels in porcine spermatozoa. (**A**) Time lag in the Ca^2+^ influx induced by 1 nM NPPC between flagellum midpiece and head of porcine capacitated spermatozoa. Images are presented in a pseudocolor forma. Arrows show Ca^2+^ fluorescence. (**B**) Fluorescence intensity of Ca^2+^ in the flagellum midpiece and head of capacitated spermatozoa, as in Figure 3A. (**C**) The percentage of spermatozoa response induced by NPPC (*n* = 3 independent experiments). (**D**) Representative micrographs of Ca^2+^ fluorescence intensity in capacitated spermatozoa after different treatments. (**E**) Comparison of the Ca^2+^ fluorescence intensity response in the spermatozoa after different treatments (*n* = 3 independent experiments). NPPC was used at 1 nM, and the inhibitor *l-cis*-Diltiazem (*l-cis*-D) was used at 50 μM with a pre-incubation of 15 sec. NNC 055-0396 (NNC), 8-Br-cGMP, BAPTA/AM, and TMB-8 were used with 2 μM, 1 mM, 5 μM, and 10 μM, respectively. Scale bars in (**A**,**D**) represent 20 μm. Bars indicate the mean ± SEM. Statistical analysis was performed by two-tailed unpaired Student’s *t*-test. *** *p* < 0.001. Each data point represents a biologically independent experiment.

We treated the capacitated spermatozoa with different concentrations of NPPC for 30 min, and then detected sperm motility using computer-assisted sperm analysis (CASA). The results showed that 1 nM NPPC significantly increased sperm motility (Table S1). In addition, NPPC significantly increased the intracellular cGMP levels of spermatozoa (Figure S1C), and the incubation of the spermatozoa with 8-bromoadenosine-cGMP (8-Br-cGMP, a cell-permeating cGMP analog), also significantly increased sperm motility (Table S2). The cGMP-sensitive CNG channels inhibitor *l-cis*-Diltiazem, but not the CatSper channel inhibitor NNC 055-0396, blocked NPPC-induced increase of sperm motility (Table S2). The activation of CNG channels can increase Ca^2+^ levels in spermatozoa [30], and Ca^2+^ signaling is required for sperm motility [31]. Thus, we examined the effect of NPPC on Ca^2+^ levels in porcine spermatozoa by measuring Ca^2+^ fluorescence intensity. The fluorescence intensity was dramatically increased in the flagellar midpiece and then in the head in response to NPPC (Figure 3A). In addition, we observed a time lag of 1 s in the response between the flagellar midpiece and the head (Figure 3A,B), consistent with our and other studies in mouse that a tail to head propagation of a calcium wave is found in response to NPPC and 8-Br-cGMP [17,30]. The initial site of Ca^2+^ elevation in porcine spermatozoa is consistent with NPR2 location. NPPC induced Ca^2+^ response in 25.0 ± 1.1% of the sperm population (Figure 3C), consistent with the percentage of capacitated spermatozoa with FAM-NPPC binding. The removal of Ca^2+^ from the medium completely blocked NPPC-induced increase in Ca^2+^ fluorescence intensity (Figure 3D,E). Furthermore, the extracellular calcium chelator bis-(o-aminophenoxy)-ethane-N,N,N,N-tetraacetic acid, tetra(acetoxymethyl)-ester (BAPTA/AM), but not intracellular calcium chelator 8-(diethylamino)octyl-3,4,5-trimethoxybenzoate hydrochloride (TMB-8), blocked NPPC-induced elevation of Ca^2+^ levels (Figure 3D,E), suggesting that NPPC-induced Ca^2+^ elevation originates from extracellular Ca^2+^ influx. We used *l-cis*-Diltiazem to determine whether CNG channels played a role in NPPC-induced Ca^2+^ elevation. The results showed that *l-cis*-Diltiazem, but not NNC 055-0396, completely blocked NPPC-induced increase in Ca^2+^ fluorescence intensity (Figure 3D,E). The incubation of spermatozoa with 8-Br-cGMP also increased Ca^2+^ fluorescence intensity (Figure 3D,E). Thus, NPPC increases porcine sperm motility and Ca^2+^ influx via cGMP-sensitive CNG channels.

### 2.4. NPPC Promotes Sperm Release from the Oviduct Isthmic Cell Aggregates

**Figure 4:**
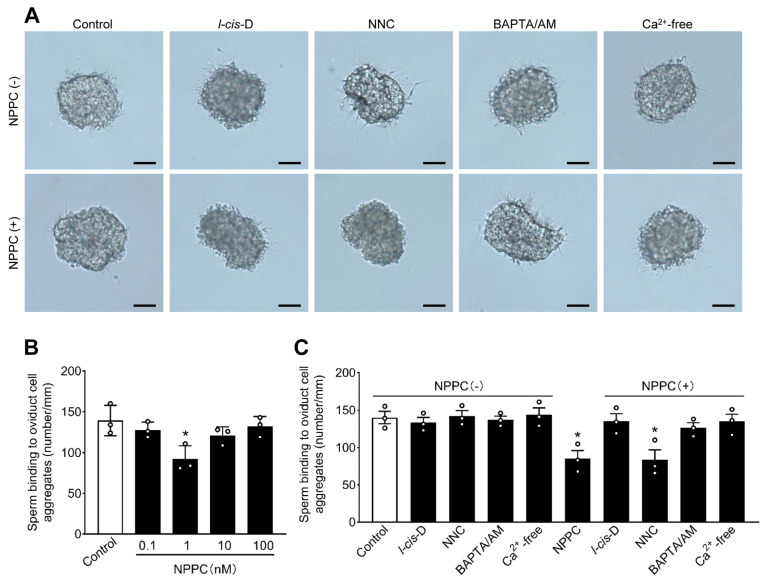
NPPC promotes sperm release from the oviduct isthmic cell aggregate. (**A**) Representative micrographs of spermatozoa-oviduct cell aggregate complexes after different treatments. Scale bars, 50 μm. (**B**) The number of spermatozoa bound to the periphery of epithelial cells was measured after incubation with different concentrations of NPPC (0.1–100 nM) for 30 min at 39 °C (*n* = 3 independent experiments). (**C**) The number of spermatozoa bound to the periphery of epithelial cells was measured after incubation with different inhibitory treatments for 30 min at 39 °C (*n* = 3 independent experiments). NPPC, *l-cis-*Diltiazem (*l-cis*-D), NNC 055-0396 (NNC), and BAPTA/AM were used with 1 nM, 50 μM, 2 μM, and 5 μM, respectively. Bars indicate the mean ± SEM. Statistical analysis was performed by two-tailed unpaired Student’s *t*-test. * *p* < 0.05. Each data point represents a biologically independent experiment.

We used an in vitro assay to test the role of NPPC in promoting porcine sperm release from the oviduct isthmic cell aggregates. This method has been used in the previous study [9]. Spermatozoa were allowed to bind the oviduct isthmic cell aggregates for 15 min, and then treated with different concentrations of NPPC. The results showed that 1 nM NPPC significantly stimulated the release of up to 34.0% of spermatozoa from the oviduct isthmic cell aggregates within 60 min of addition (Figure 4A,B). The pre-incubation of spermatozoa with *l-cis*-Diltiazem, but not NNC 055-0396, completely blocked NPPC-induced spermatozoa release from the oviduct isthmic cell aggregates (Figure 4A,C). Furthermore, the removal of Ca^2+^ from the medium, or the addition of extracellular calcium chelator BAPTA/AM could also completely block NPPC-induced sperm release (Figure 4A,C). These results suggest that NPPC induces porcine sperm release from oviduct isthmic cells via cGMP-sensitive CNG channels and Ca^2+^ influx.

### 2.5. The Mature COCs Promote NPPC Expression in the Ampullae

**Figure 5:**
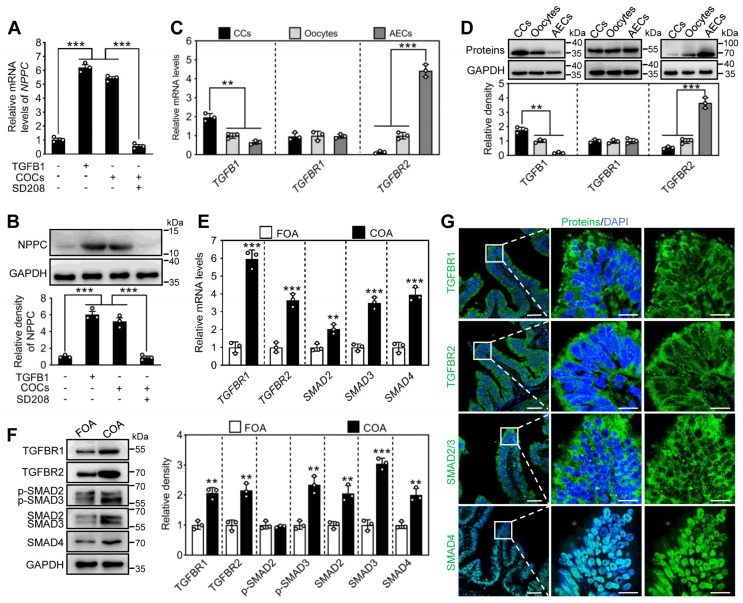
The mature COCs promote ampullary NPPC expression. (**A**,**B**) The effect of mature COCs, TGFB1 (10 ng/mL) and SD208 (1 μM) on NPPC mRNA (**A**) and protein (**B**) expression in ampullary epithelial cells (n = 3 independent experiments). (**C**,**D**) Comparison of steady-state mRNA (**C**) and protein (**D**) levels of TGFB1, TGFBR1, and TGFBR2 in the oocytes, cumulus cells, and ampullary epithelial cells (n = 3 independent experiments). (**E**) Comparison of steady-state levels of *TGFBR1*, *TGFBR2*, *SMAD2*, *SMAD3*, and *SMAD4* mRNA in the ampullae at follicular and corpus rubrum phase (n = 3 independent experiments). (**F**) The protein levels of TGFBR1, TGFBR2, p-SMAD2, p-SMAD3, SMAD2, SMAD3, and SMAD4 in the ampullae at follicular and corpus rubrum phase (n = 3 independent experiments). GAPDH was used as an internal control. (**G**) Immunofluorescence stain of TGFBR1, TGFBR2, SMAD2/3, and SMAD4 (green) in the ampullae at the corpus rubrum phase. The nuclei were counterstained by DAPI (blue). The amplified views of the boxed area are shown on the right-hand side. Scale bars, 50 μm. Representative examples of three independent experiments are shown. Bars indicate the mean ± SEM. Statistical analysis was performed by two-tailed unpaired Student’s *t*-test. ** *p* < 0.01 and *** *p* < 0.001. Each data point represents a biologically independent experiment. CCs, cumulus cells; AECs, ampullary epithelial cells; FOA, oviductal ampullae at follicular phase; COA, oviductal ampullae at corpus rubrum phase.

We further investigated the molecular mechanism of NPPC expression in pig. After ovulation, the mature COCs reside in the ampullae for fertilization. Here, immature COCs were collected from the antral follicles of porcine ovaries, and cultured with EGF for 44 h to acquire the mature COCs [32]. The mature COCs, but not the immature COCs, promoted NPPC expression in the ampullae (Figure 5A,B and Figure S2). The mature COC-induced NPPC was completely blocked by the TGFBR1 inhibitor SD208 (Figure 5A,B), indicating that COCs promote NPPC expression in the ampullae via TGF-β signaling. Similarly, the addition of TGFB1 significantly increased NPPC expression in the ampullae, which was blocked by SD208 (Figure 5A,B). TGFBR1 can be also activated by oocyte paracrine factors, such as growth differentiation factor 9 (GDF9) and bone morphogenetic protein 15 (BMP15) [27]. However, the denuded oocytes (DO) could not upregulate ampullary NPPC mRNA levels (Figure S2), suggesting that TGF-β signaling from cumulus cells promotes NPPC expression.

Subsequently, we examined the expression pattern of TGF-β ligands and receptors in the mature COCs and ampullary epithelial cells. In cumulus cells, oocytes, and ampullary epithelial cells, the relative abundance of *TGFB1* mRNA was much higher than that of *TGFB2* and *TGFB3* mRNAs (Figure S3). Moreover, TGFB1 mRNA and protein levels in cumulus cells of the mature COCs were significantly higher than those in oocytes and ampullary epithelial cells (Figure 5C,D). TGFBR2 mRNA and protein levels in ampullary epithelial cells were significantly higher than those in the cumulus cells and oocytes (Figure 5C,D). These results indicate that TGFB1 is mainly expressed in cumulus cells, whereas TGFBR2 is mainly expressed in ampullary epithelial cells.

We compared the levels of TGFBRs and their downstream signaling molecules in the ampullary epithelial cells from follicular and corpus rubrum phase oviducts. The mRNA and/or protein levels of TGFBR1, TGFBR2, SMAD2, SMAD3, SMAD4, and p-SMAD3 were significantly higher in the ampullary epithelial cells from the corpus rubrum phase oviducts than those from the follicular phase oviducts (Figure 5E,F). Immunofluorescent staining results showed that TGFBR1, TGFBR2, and SMAD2/3 were located in the cytoplasm and SMAD4 in the nucleus of the ampullary epithelial cells (Figure 5G).

### 2.6. EGF Promotes TGFB1 Expression in Cumulus Cells

**Figure 6:**
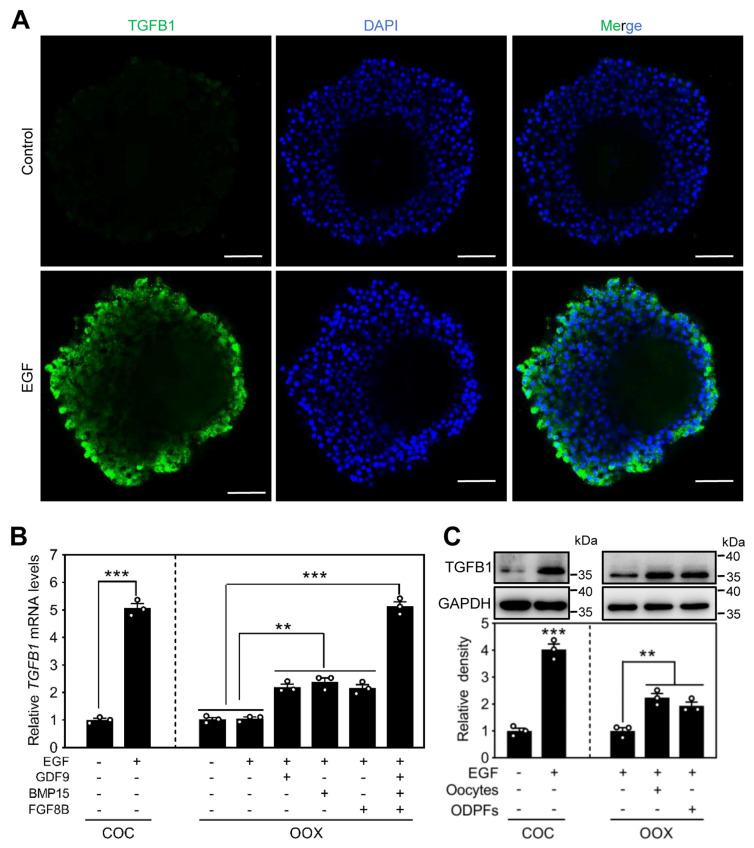
EGF promotes TGFB1 expression in cumulus cells. (**A**) Immunofluorescence staining of TGFB1 (green) in the COCs. The immature COCs from 3–6 mm follicles were cultured without or with EGF (10 ng/mL) for 44 h. The nuclei were counterstained by DAPI (blue). Scale bars, 50 μm. Representative examples of three independent experiments are shown. (**B**,**C**) The effects of EGF, oocytes, and oocyte paracrine factors (ODPFs) on TGFB1 mRNA (**B**) and protein (**C**) expression in cumulus cells. The immature COCs or OOX cumulus cells were cultured in the medium, supplemented with EGF (10 ng/mL), human GDF9 (500 ng/mL), human BMP15 (500 ng/mL), and/or human FGF8B (FGF8, 100 ng/mL) for 44 h. ODPFs, GDF9 + BMP15 + FGF8B. Bars indicate the mean ± SEM. Statistical analysis was performed by two-tailed unpaired Student’s *t*-test. ** *p* < 0.01 and *** *p* < 0.001. Each data point represents a biologically independent experiment.

The maturation of COCs was induced by LH-EGFR signaling in many species, including pig [10]. We cultured the immature COCs with EGF to examine the effect of EGFR signaling on TGFB1 expression in cumulus cells. Compared to the control, EGF obviously increased the cytoplasm accumulation of TGFB1 in cumulus cells (Figure 6A). Consistent with this, EGF significantly increased the mRNA and protein levels of TGFB1 in cumulus cells (Figure 6B,C). EGF-promoted TGFB1 mRNA levels were completely blocked by EGFR inhibitor AG1478 (Figure S4). However, EGF could not increase the mRNA levels of GDF9 and BMP15 (Figure S4). These results indicate that the expression of TGFB1 in porcine cumulus cells is increased during EGFR signaling-induced maturation of COCs.

Generally, EGFR signaling-induced cumulus cell gene expression requires the participation of oocyte paracrine factors [33]. We found that EGF could not increase TGFB1 mRNA and protein levels in porcine cumulus cells of oocytectomized (OOX) complexes (Figure 6B,C), suggesting that oocyte paracrine factors are required for EGF-induced TGFB1 expression in cumulus cells. GDF9, BMP15, and fibroblast growth factor 8B (FGF8) are paracrine growth factors secreted by oocytes. In the presence of EGF, each of these three paracrine factors alone significantly promoted TGFB1 mRNA levels in OOX cumulus cells (Figure 6B), and the combination of all three factors promoted TGFB1 mRNA levels in OOX cumulus cells equivalent to those in intact COC cumulus cells (Figure 6B). Consistent with this, Western blotting results showed that both oocytes and the paracrine factors promoted TGFB1 protein levels in OOX cumulus cells in the presence of EGF (Figure 6C). These results indicate that EGFR signaling and cooperation with oocyte paracrine factors promotes TGFB1 expression in porcine cumulus cells.

## 3. Discussion

The present study showed that NPPC was mainly expressed in oviduct ampullary epithelial cells in pig, whereas its cognate receptor NPR2 was located on the midpiece of sperm flagellum, consistent with our previous study in mouse [17]. Moreover, the mature COCs promoted NPPC expression in ampullary epithelial cells via TGF-β signaling, and NPPC increased sperm motility and induced sperm release from oviduct isthmic cells via cGMP-sensitive CNG channel-dependent Ca^2+^ influx.

We failed to obtain the results of immunofluorescent staining with the available NPR2 antibody. Thus, we used FAM-NPPC to bind the receptor NPR2, and the positive rate was low in the capacitated spermatozoa. The possible reason is that only the spermatozoa with exposed NPR2 could be bound by FAM-NPPC. In mice, only a small fraction of spermatozoa is capacitated at a given time [34] to prolong the sperm lifespan for fertilization. The capacitation of spermatozoa may result in unmasking NPR2, which is a prerequisite for spermatozoa in response to NPPC. A similar mechanism may exist in pig. NPPC induced Ca^2+^ influx and the increase of motility in porcine spermatozoa via CNG channels. NPPC also promoted sperm release from oviduct isthmic cell aggregates via cGMP-sensitive CNG channels and Ca^2+^ influx. Thus, CNG channel-dependent Ca^2+^ influx may contribute to NPPC-promoted porcine sperm motility [35,36,37], and then provide the force necessary for overcoming the adhesion between the spermatozoa and isthmic epithelium [9]. On the other hand, we cannot exclude that NPPC promotes sperm release by stimulating the enzyme activity to degrade the adhesion.

Consistent with our previous study in mouse [17], porcine mature COCs promoted NPPC expression in the ampullae. The mature COC-induced NPPC was blocked by TGFBR1 inhibitor. TGFBR1 can be activated not only by TGFB ligands binding to TGFBR2 [25], but also by oocyte paracrine factors (GDF9 and BMP15) binding to ACVR2B receptor [27]. However, the denuded oocytes from mature COCs could not increase ampullary *NPPC* mRNA levels, suggesting that GDF9 and BMP15 derived from the oocytes cannot promote NPPC expression in the ampullae. Interestingly, the immature COCs could not induce *NPPC* expression in the ampullae. The maturation of COCs is induced by LH-activated EGFR signaling in cumulus cells [10,11]. In our study, the activation of EGFR by EGF promoted TGFB1 expression in cumulus cells, and the addition of TGFB1 promoted NPPC expression in the ampullae. Thus, TGFBs, especially TGFB1 from mature COCs, promote NPPC expression in the ampullae. This is consistent with our and others’ studies which found that TGF-β signaling promotes NPPC expression in various cell types [19,23,33]. Additionally, the mRNA and protein levels of TGFBRs and their downstream molecules were significantly higher in the ampullae at corpus rubrum phase (after ovulation) than those at follicular phase (before ovulation). The increase of these proteins in the ampullae at corpus rubrum phase is beneficial for mature COC-promoted NPPC expression in the ampullae when ovulation occurs in pig. The phosphorylation levels of SMAD3, not SMAD2, were significantly increased in porcine corpus rubrum phase oviducts, consistent with our previous study in mouse granulosa cells [23]. SMAD3 can bind to *Nppc* promoter to enhance *Nppc* expression [23]. Thus, p-SMAD3 may be the factor to achieve TGF-β signal transduction into the nucleus and initiate the transcription of *NPPC* in porcine ampullae. On the other hand, the increase of p-SMAD2 in the porcine ampullae was not detected, possibly due to the time limit of sample collection. In mouse, ovulated COCs promote NPPC expression in the ampullae by binding cumulus cell-derived TGFB1 to TGFBR in the ampullary epithelial cells, which is critical for sperm migration for fertilization [33]. A similar mechanism may exist in pig: mature COCs induce NPPC expression in the ampullae via TGF-β signaling, and then NPPC diffuses to the isthmus for sperm release.

EGF, cooperation with oocyte paracrine factors, promoted TGFB1 expression in cumulus cells of COCs, consistent with our recent report in mouse [33]. EGFR signaling-activated cyclic adenosine 3′,5′-monophosphate (cAMP) response element-binding protein (CREB) in mouse cumulus cells, together with oocyte paracrine factor-activated SMAD2/3, induce expansion-related gene expression and consequent cumulus expansion [38]. In the present study in pig, EGFR signaling may activate CREB, and then cooperate with oocyte paracrine factor-activated SMAD2/3 to promote TGFB1 expression in cumulus cells. Thus, oocytes are involved in porcine sperm release by promoting TGFB1 expression in cumulus cells and then NPPC expression in the ampullae. The high expression of NPPC in the ampullae at corpus rubrum phase may be the reason for the ovulation-triggered sperm release. In addition, TGF-β ligands, particularly TGFB1, also present in the porcine oviduct ampullae, consistent with a previous report on human and mouse [39,40]. TGF-β in the oviductal microenvironment may promote a small amount of NPPC expression, resulting in a small amount of sperm release from the isthmic reservoir before ovulation [41]. The sequence of peptide NPPC is highly conserved [17]. NPPC promoted porcine sperm release from the oviduct isthmic cell aggregates, consistent with our recently study that NPPC promotes mouse sperm migration from the isthmic reservoir to the ampulla for fertilization [33]. This suggests that the function of NPPC may be highly conserved in mammals in promoting sperm release from the isthmic reservoir to the ampullae for fertilization.

**Figure 7:**
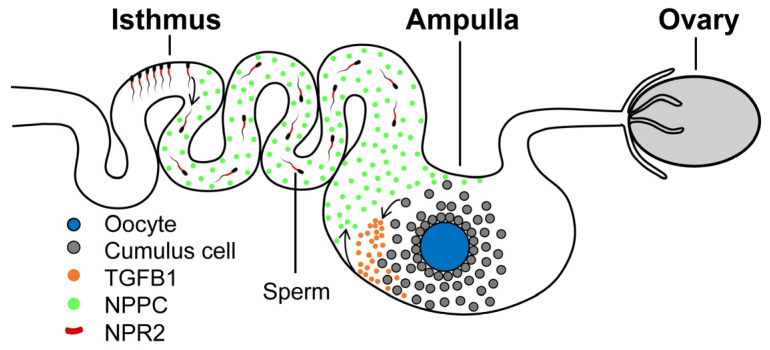
Model showing that mature porcine COC induced NPPC in the ampulla for sperm release. The mature porcine COCs promote the expression of NPPC in the ampullae, and then NPPC promotes sperm release from the isthmic reservoir for fertilization when ovulation occurs.

We hypothesize that porcine mature COCs promote the expression of NPPC in the ampullae and then promote sperm release from the isthmic reservoir for fertilization when ovulation occurs (Figure 7).

## 4. Materials and Methods

### 4.1. Chemicals and Culture Medium

The reagents used in this study, unless otherwise stated, were purchased from Sigma-Aldrich (St. Louis, MO, USA). The basic medium used for COC and oviductal ampulla culture was TCM199 medium (Thermo Fisher Scientific Corporation, Waltham, MA, USA) supplemented with 0.23 mM sodium pyruvate, 2 mM glutamine, 3 mg/mL lyophilized crystalline BSA, 100 IU/mL penicillin G, and 25 mg/mL streptomycin sulfate.

### 4.2. Collection and Processing of Spermatozoa

Porcine semen was provided by Guangxi Yangxiang Co., Ltd. (Guigang, China). The semen samples were stored at 16 °C to 18 °C for 24 h before use. The pooled extended semen of 3 mL was washed through a Percoll cushion containing 5.4 mL Percoll, 4 mL dmTALP (2.1 mM CaCl_2_, 3.1 mM KCl, 1.5 mM MgCl_2_, 100 mM NaCl, 0.29 mM KH_2_PO_4_, 0.36% lactic acid, 25 mM NaHCO_3_, 0.6% BSA, 1 mM pyruvic acid, and 20 mM HEPES), and 0.6 mL 10× HBS (1.3 M NaCl, 40 mM KCl, 10 mM CaCl_2_, 5 mM MgCl_2_), and centrifuged for 10 min at 800× *g*. Then, the spermatozoa were pelleted again for 5 min at 500× *g* after washing with dmTALP medium. Finally, the concentration of spermatozoa was determined with a hemocytometer and adjusted according to the experiment. The spermatozoa were cultured in dmTALP medium for 1.5 h at 39 °C for capacitation. For protein assay, the heads and tails from 3 × 10^8^ capacitated spermatozoa were separated according to our previous study [17]. Briefly, the spermatozoa were snapped frozen in liquid nitrogen and diluted with 600 μL of phosphate-buffered saline (PBS). The sperm suspension was passed fifty times through a 26-gauge needle on ice and centrifuged at 250× *g* for 3 min at 4 °C to precipitate the sperm heads. Sperm flagella were recovered from the supernatant by centrifugation at 10,000× *g* for 10 min at 4 °C.

### 4.3. Identification and Localization of NPR2

The location of NPR2 in spermatozoa was identified by FAM-NPPC (the mono-5- (and 6)-carboxyfluorescein label NPPC. Phoenix Pharmaceuticals, Belmont, CA, USA). The spermatozoa without or with capacitation were incubated with 100 nM FAM-NPPC for 30 min at 39 °C to ensure full reaction. The spermatozoa were then fixed with 2% paraformaldehyde for 5 min, and washed three times with PBS to remove the unbound ligand. The fluorescent-labeled ligand binding was assessed by LSM 800 confocal microscope (Zeiss, Oberkochen, Germany). The spermatozoa with uneven dye loading were excluded from the analysis. For competition experiments, the capacitated spermatozoa were co-incubated with 100 nM FAM-NPPC and 1 μM unlabeled NPPC for 30 min at 39 °C.

### 4.4. Sperm Motility

The capacitated spermatozoa were incubated in dmTALP medium, supplemented with different concentrations of NPPC (0.01–10 nM), NNC 055-0396 (CatSper channel inhibitor, 2 μM), 8-Br-cGMP (1 mM), and/or *l-cis*-Diltiazem (CNG channels inhibitor, 50 μM) for 30 min. The sperm motion was detected using a CASA system (Version 12 CEROS, Hamilton Thorne Research, Beverly, MA, USA). The parameters included straight-line velocity (VSL), amplitude of lateral head displacement (ALH), average path velocity (VAP), curvilinear velocity (VCL), beat cross frequency (BCF), and percentage of linearity (LIN, VSL/VCL × 100%). For each experimental condition, five random fields were evaluated for a minimum total of 100 cells in each field.

### 4.5. Imaging Analysis of the Intracellular Ca^2+^ Levels

Ca^2+^ levels in the capacitated spermatozoa were detected according to the previous study [18]. Briefly, the capacitated spermatozoa were incubated with 5 μM Fluo 3-AM (Dojindo Laboratories, Kumamoto, Japan) and 0.06% pluronic F-127 for 30 min at 39 °C in the dark. The capacitated spermatozoa (3–5 × 10^5^ cells/mL) were placed on glass coverslips treated with polylysine at 0.01% in the recording chamber for 5 min, and then the external solution was infused to wash the supernatant. Imaging analysis of Ca^2+^ response in motile spermatozoa was monitored using a LSM 800 confocal microscope (Zeiss, Oberkochen, Germany). We used a sample frequency of 1 Hz, and recorded Ca^2+^ fluorescence intensity for 3–4 min after different treatments. All Ca^2+^ imaging experiments were carried out at 39 °C. Cells with uneven dye loading were excluded from the analysis. NPPC was used at 1 nM, and the inhibitor *l-cis*-Diltiazem was used at 50 μM with a pre-incubation of 15 sec. NPPC and the inhibitor were dropped into the recording chamber using pipette tips and the recordings were conducted in the continuous presence of stimuli. Ca^2+^-free experiments were conducted using Ca^2+^-free modified Tris-buffered medium (mTBM) without BSA, obtained by omitting Ca^2+^ and adding 1 mM EGTA. NNC 055-0396, 8-Br-cGMP, BAPTA/AM, and TMB-8 were used with 2 μM, 1 mM, 5 μM, and 10 μM, respectively. The highest level of Ca^2+^ fluorescence intensity in at least 50 spermatozoa was used for the presentation of Ca^2+^ level of spermatozoa in different treatment. Each experiment was repeated three times.

### 4.6. Measurement of cGMP Levels in Spermatozoa

The capacitated spermatozoa of 100 μL (1 × 10^7^ cells/mL) were incubated without or with 1 nM NPPC for 20 min, and then collected and solubilized in 100 μL of 0.1 M HCl on ice for 15 min. The samples were thawed and centrifuged for 5 min at 12,000× *g*, and the supernatant was collected and dried in an oven at 60 °C for cGMP assay. The levels of cGMP were determined by a cGMP-enzyme immunoassay kit (Cayman Chemical, Ann Arbor, MI, USA) according the manufacturer’s instruction.

### 4.7. Preparation of Oviduct Isthmic Epithelial Cell Aggregates

Porcine oviducts were collected from a local abattoir and transported to the laboratory within 2 h at 4 °C in PBS. Spherical aggregates were prepared as previously described with slightly modification [9]. Briefly, the isthmic epithelial cells were stripped from the oviductal isthmus using the edge of the microscope slide. The isthmic epithelial cells in PBS were transferred into a 15 mL conical tube and centrifuged at 100× *g* for 1 min. After removing the supernatant, the cells were disaggregated in 15 mL PBS carefully and centrifuged again. After adjusting the volume of the isthmic epithelial cells to 12 mL with dmTALP medium, the cells were divided evenly into three culture dishes. The isthmic epithelial cells were allowed to re-aggregate for 90 min at 39 °C, and the spherical aggregates (100–150 µm in diameter) were selected for testing sperm release.

### 4.8. Assay of Sperm Binding to and Release from the Oviduct Isthmic Cell Aggregates

Assay of sperm binding to and release from oviduct isthmic cell aggregates was conducted according to the previous report [9]. Briefly, the spherical oviduct isthmic cell aggregates were washed twice in fresh dmTALP medium. Then, 2 µL spermatozoa at a final concentration of 5 × 10^5^ cells/mL were added into a 50 µL droplet containing 10 oviduct isthmic cell aggregates. Spermatozoa and the oviduct isthmic cell aggregates were incubated at 39 °C for 15 min for sperm binding to form the complexes. Then, the complexes were incubated without or with 1 nM NPPC for 30 min at 39 °C. In some experiments, the complexes were pre-incubated with *l-cis*-Diltiazem (50 µM), NNC 055-0396 (2 μM), or BAPTA/AM (5 μM) for 5 min at 39 °C, and then incubated without or with NPPC. At the end of incubation, the free and loosely attached spermatozoa were removed from the oviduct isthmic cell aggregates by washing with dmTALP medium. The aggregates were fixed with 4% paraformaldehyde for 10 min and then transferred to a 3 µL droplet in the microscope slide. Images were captured using Axio Vert. A1 microscope (Zeiss, Oberkochen, Germany). The number of spermatozoa bound to the periphery of each aggregate was enumerated, and the circumference of the aggregate was calculated using Labscope V 3.2 software (Zeiss, Oberkochen, Germany). The number of spermatozoa bound per millimeter of circumference was calculated for each aggregate, and the average number of spermatozoa from 10 aggregates was used for statistical analysis. All the cultures were incubated at 39 °C under 5% CO_2_.

### 4.9. Culture of Ampullae, COCs, OOXs, and Oocytes

Porcine ovaries were collected from a local abattoir and transported to our laboratory within 2 h in PBS at 4 °C. The immature COCs were aspirated from the follicles with 3–6 mm in diameter, and then 100–120 COCs were cultured in 500 μL TCM-199 medium with EGF (10 ng/mL) for 44 h to acquire the mature COCs. The denuded oocytes (DOs) were obtained by removing the cumulus cells from the mature COCs with 1 mg/mL hyaluronidase. One ampulla at follicular phase was cultured in 200 μL TCM-199 medium, supplemented with 150 immature or mature COCs, 150 DOs, 10 ng/mL TGFB1, and/or 1 μM SD208 (TGFBR1 inhibitor) for 3 h. The ampullae were collected at the end of culture, and the epithelial cells were scraped by the edge of a microscope slide. In some experiments, the epithelial cells were isolated from the ampullae at follicular and corpus rubrum phases. The samples were stored at -80 °C for mRNA and protein analysis.

OOX cumulus cells were produced by microsurgically removing the oocytes, but not the zona pellucida, from the immature COCs. Groups of 100–120 COCs or OOX cumulus cells were cultured in the medium, supplemented with EGF, growth differentiation factor 9 (GDF9, 500 ng/mL, human), bone morphogenetic protein 15 (BMP15, 500 ng/mL, human), and/or fibroblast growth factor 8B (FGF8, 100 ng/mL, human) in 500 μL medium. After 44 h culture, cumulus cells were collected for gene and protein analysis. All the cultures were incubated at 39 °C under 5% CO_2_.

### 4.10. Western Blotting

The samples were collected from 3 × 10^8^ sperm head and tails, 200 oocytes, the cumulus cells from 100 COCs or OOXs, and the ampullary epithelial cells from one ampulla. Total protein was extracted in WIP buffer (Cell Chip Biotechnology, Beijing, China) with 1 mM phenylmethylsulfonyl fluoride on ice. Normalized protein amounts were separated by 10% SDS-PAGE and then transferred to polyvinylidene fluoride membranes (Millipore, Billerica, MA, USA). The membranes were incubated with primary antibodies (Table S3) overnight at 4 °C after blocking with 5% nonfat milk in Tris-buffered saline (TBS) for 2 h at room temperature. Then, the membranes were washed three times each for 5 min in TBS with 0.1% Tween-20, and incubated with horseradish peroxidase conjugated secondary antibodies (each diluted 1:5000, ZSGB-BIO, Beijing, China). The blots were detected using the SuperSignal West Pico Kit (Thermo Fisher Scientific Corporation, Waltham, MA, USA) and visualized by the Tanon 5200 chemiluminescent imaging system (Tanon, Shanghai, China). GAPDH was used as an internal control. The uncropped, unedited blots are shown in Figure S5.

### 4.11. RNA Isolation and qRT-PCR

Total RNA of porcine spermatozoa, ampullary epithelial cells, oocytes, and cumulus cells was extracted using the RNeasy micro-RNA isolation kit (Qiagen, Valencia, CA, USA) according to the manufacturer’s instructions. RNA (no more than 1 μg) was reverse transcribed into cDNA using the QuantiTect Reverse Transcription System (Qiagen, Valencia, CA, USA). qRT-PCR was conducted using a Light Cycler 96 instrument (Roche, Basel, Switzerland). Relative gene expression was quantified using the threshold cycle value and normalized using *GAPDH* as a housekeeping gene. Primer sequences are listed in Supplementary Table S4.

### 4.12. Immunohistochemistry and Immunofluorescence Analysis

Oviductal ampullae at corpus rubrum phase were fixed in cold 4% paraformaldehyde overnight at 4 °C, dehydrated in gradient alcohol, embedded in paraffin, and sectioned at 5 μm. Oviductal ampulla sections were deparaffinized, rehydrated, and subjected to antigen retrieval with 0.01% sodium citrate buffer (pH 6.0) at high temperature (95–98 °C) for 16 min. The sections were rinsed thoroughly with PBS and blocked with 10% normal donkey serum in PBS for 1 h at room temperature. The COCs were fixed in 4% paraformaldehyde for 2 h at 4 °C and blocked with 10% normal donkey serum in PBS for 1 h at room temperature. After blocking, all the samples were incubated with primary antibodies (Table S3) overnight at 4 °C, and then washed three times in PBS and incubated with Alexa Fluor 488-conjugated secondary antibodies (1:200, Thermo Fisher Scientific Corporation, Waltham, MA, USA) for 1 h at 37 °C. Finally, the samples were stained with 4′,6-diamidino2-phenylindole (DAPI) for 5 min to counterstain the nuclei. The isotype-specific immunoglobulins (IgG) at the same protein concentration as the primary antibodies was used for the negative control (Abcam, ab172730, Cambrige, UK). Immunohistochemistry was performed using Histostain™-SP Kits (PV-9001, ZSGB-BIO) and DAB peroxidase substrate kits (ZLI-9017, ZSGB-BIO) according to the manufacturer’s instruction. The staining was examined using a Zeiss LSM 800 confocal microscope (Zeiss).

### 4.13. Statistical Analysis

All experiments were carried out at least three times. The data were analyzed and graphed using GraphPad Prism software (version 8.3, La Jolla, CA, USA). The statistical significance between two groups was analyzed by two-tailed unpaired Student’s *t*-tests, and *p* < 0.05 was considered significant.

## Data Availability

All data generated or analyzed during this study are available from the corresponding author on reasonable request.

## Supplementary Materials

The following supporting information can be downloaded at: https://www.mdpi.com/article/10.3390/ijms24043118/s1.
