# Supplementary material for: The Mature COC Promotes the Ampullary NPPC Required for Sperm Release from Porcine Oviduct Cells
Source: Int J Mol Sci. 2023 Feb 4;24(4):3118. doi: 10.3390/ijms24043118 (PMC9967908; doi:10.3390/ijms24043118)
Supplement: Supplementary file 1 [file ijms-24-03118-s001.zip › ijms-2126276-supplementary.pdf]

Supplemental Material

The Mature COC Promotes the Ampullary NPPC Required for Sperm Release from Porcine Oviduct Cells

Zhanying Wu, et al.

Supplementary Figures and Figure Legends

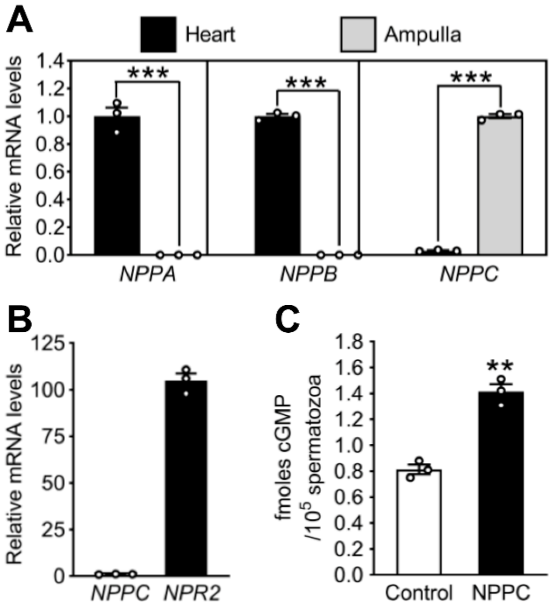

Figure S1. **NPPC expression and the cGMP levels in porcine spermatozoa**. (**A**) The mRNA levels of *NPPA*, *NPPB* and *NPPC* in the ampulla and heart (n = 3 independent experiments). Heart is the positive tissue for NPPA, NPPB and NPPC. (**B**) The mRNA levels of *NPPC* and *NPR2* in fresh porcine spermatozoa (n = 3 independent experiments). (**C**) Capacitated spermatozoa were incubated without or with 1 nM NPPC for 20 min, and the amounts of cGMP were measured using enzyme immunoassay kits. Bars indicate the mean ± SEM. n = 3 independent experiments. Statistical analysis was performed by two−tailed unpaired Student's *t*−test. ***P < 0.001 and **P < 0.01. Each data point represents a biologically independent experiment.

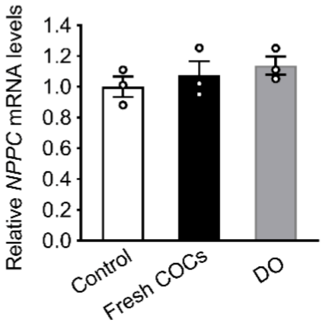

Figure S2. **The effect of fresh COCs or denuded oocytes on *NPPC* mRNA levels in the ampullary epithelial cells**. The ampullae isolated from oviducts at follicular phase were co−cultured with fresh COCs (immature COCs. 150 COCs/200 μL) or denuded oocytes (Dos, 150 oocytes/200 μL) for 3 h. At the end of the culture, ampullary epithelial cells were scraped using the edge of a microscope slide and used for *NPPC* mRNA measurement. Bars indicate the mean ± SEM. n = 3 independent experiments. Each data point represents a biologically independent experiment.

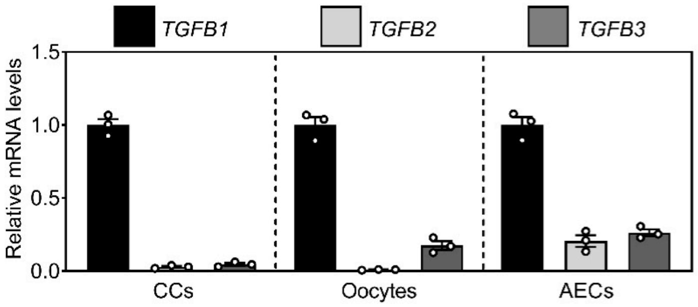

Figure S3. **Comparison of steady−state levels of *TGFB1*, *TGFB2*, and *TGFB3* mRNA in cumulus cells**, **oocytes**, **and ampullary epithelial cells**. Bars indicate the mean ± SEM. n = 3 independent experiments. Each data point represents a biologically independent experiment.

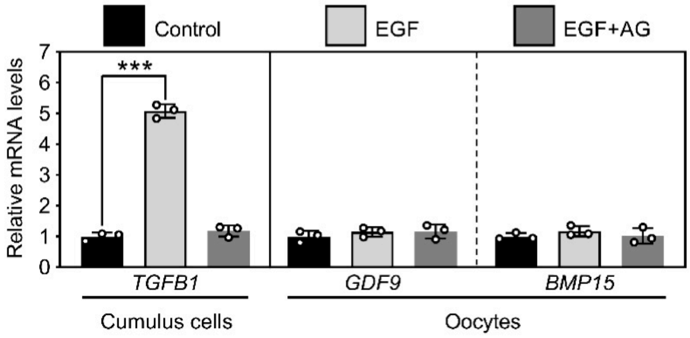

Figure S4. **The effect of EGF on the mRNA levels of *TGFB1*, *GDF9* and *BMP15* in COCs**. Immature COCs were cultured with EGF (10 ng/mL) and/or AG1478 (1 μM). n = 3 independent experiments. Statistical analysis was performed by two−tailed unpaired Student's *t*−test. ***$P < 0.001$. Each data point represents a biologically independent experiment. AG, AG1478, EGFR inhibitor.

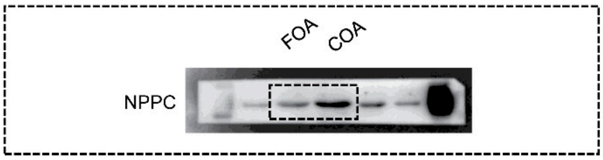

Fig. 1A

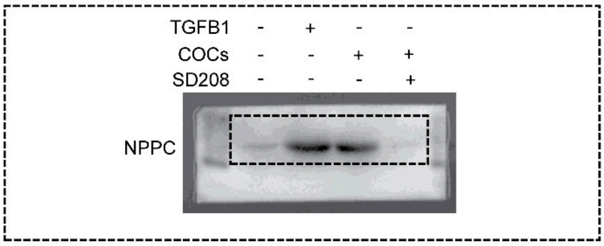

Fig. 5B

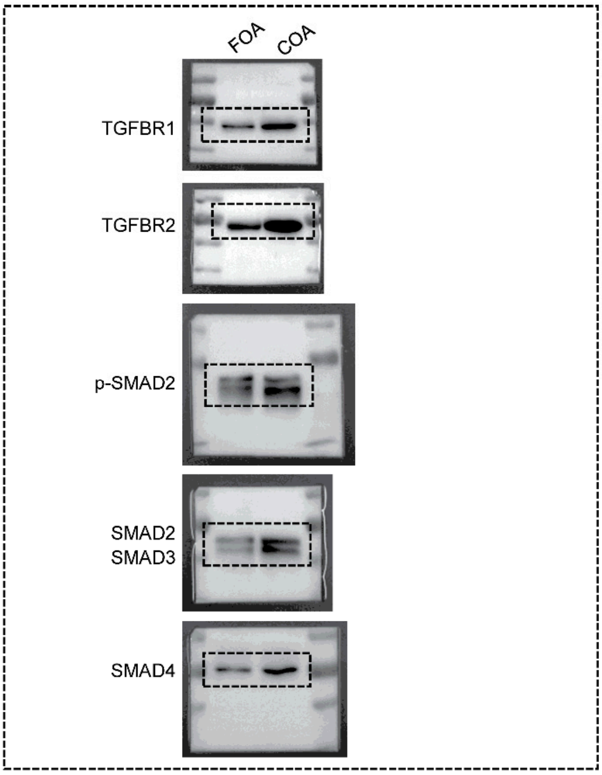

Fig. 5F

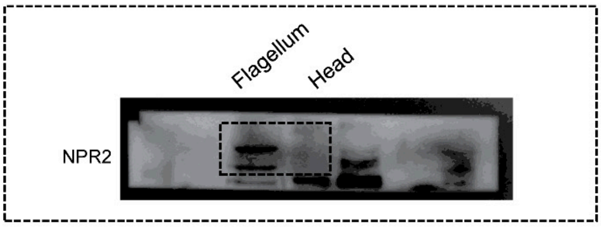

Fig. 2B

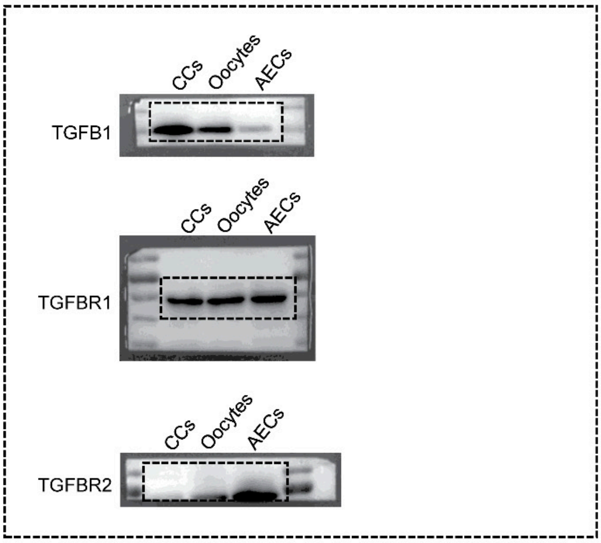

Fig. 5D

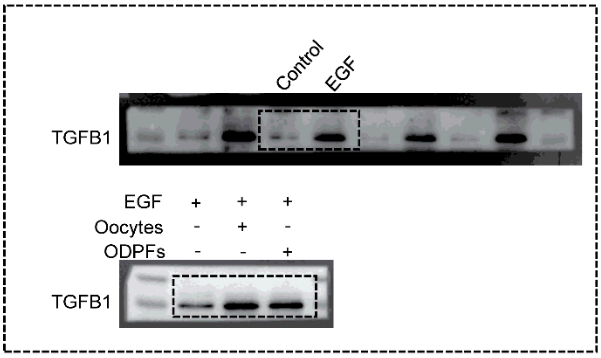

Fig. 6C

Figure S5. **Uncropped scans of the Western blotting results**.

Tables

Table S1. **The effect of NPPC on sperm motility**.

	Control	NPPC (0.01 nM)	NPPC (0.1 nM)	NPPC (1 nM)	NPPC (10 nM)
Motility%	60.1 ± 3.9	63.1 ± 3.7	66.11 ± 4.8	77 ± 3.1*	61.1 ± 4.3
VAP (μm/s)	72.66 ± 7.9	73.3 ± 7.6	75.9 ± 8.2	77.4 ± 12.0	73.6 ± 5.9
VSL (μm/s)	45.1 ± 6.5	45.9 ± 2.6	46.2 ± 3.7	47.2 ± 6.9	46.9 ± 3.9
VCL (μm/s)	152.4 ± 8.8	158.1 ± 6.3	157.6 ± 4.9	161.5 ± 10.2	155.3 ± 8.3
ALH	8.5 ± 0.8	9.0 ± 0.7	8.9 ± 0.6	9.0 ± 0.6	8.6 ± 0.9
BCF	36.9 ± 1.0	38.7 ± 2.1	37.2 ± 2.8	36.5 ± 1.5	37.4 ± 1.5
Straightness%	59.5 ± 4.7	60.5 ± 5.8	59.2 ± 4.8	58.1 ± 7.1	61.4 ± 6.7
Linearity%	28.5 ± 2.5	28.6 ± 3.5	28.4 ± 2.3	28.9 ± 3.1	28.8 ± 2.6
Elongation%	39.4 ± 1.9	38.4 ± 2.9	41.3 ± 5.9	39.0 ± 4.5	39.7 ± 4.1
Area μm/sq	17.3 ± 2.1	18.2 ± 3.2	17.5 ± 3.6	17.2 ± 2.8	18.0 ± 1.9

The motility parameters of spermatozoa were detected after incubation with different concentrations of NPPC. VAP, average path velocity; VSL, straight-line velocity; VCL, curvilinear velocity; ALH, amplitude of lateral head displacement; BCF, beat cross frequency. Bars indicate the mean ± SEM of three experiments. n = 500 for each sample. $^*P < 0.05$.

Table S2. **Sperm motility parameters were determined after incubation with NPPC, 8−Br−cGMP, *l*−*cis*−D and NNC 55−0396**.

Treatments	Control	NPPC (1 nM)	8−Br−cGMP	*l*−*cis*−D	NNC 55−0396
Motility%	60.1 ± 2.6	76.9 ± 4.1*	75 ± 5.7*	61.0 ± 4.1	76.0 ± 3.6*
VAP (μm/s)	72.66 ± 7.9	77.1 ± 12.0	75.7 ± 11.7	69.0 ± 6.2	75.5 ± 12.3
VSL (μm/s)	45.1 ± 6.5	47.9 ± 6.9	46.8 ± 8.8	46.8 ± 7.6	46.7 ± 4.3
VCL (μm/s)	152.4 ± 8.8	161.7 ± 10.2	158.7 ± 11.5	154.8 ± 12.5	159.3 ± 18.1
ALH	8.5 ± 0.8	8.9 ± 0.7	8.7 ± 1.0	8.1 ± 0.8	8.8 ± 0.8
BCF	36.9 ± 1.0	36.6 ± 1.7	35.4 ± 1.9	35.1 ± 1.4	35.7 ± 1.3
Straightness%	58.5 ± 4.7	58.0 ± 7.2	56.4 ± 5.2	56.5 ± 3.2	57.0 ± 4.8
Linearity%	28.5 ± 2.5	28.0 ± 3.5	27.2 ± 5.5	27.1 ± 5.1	27.5 ± 4.5
Elongation%	39.4 ± 1.9	39.0 ± 4.7	37.8 ± 2.4	38.4 ± 2.8	38.2 ± 1.7
Area μm/sq	17.3 ± 2.1	17.0 ± 2.9	16.7 ± 4.9	16.4 ± 4.5	16.9 ± 3.9

Motility parameters of spermatozoa were detected after different treatment. The capacitated spermatozoa were incubated in dmTALP medium, supplement with different concentrations of NPPC (0.01-10 nM), NNC 55−0396 (NNC, 2 μM), 8−Br−cGMP (1 mM), and/or *l*−*cis*−Diltiazem (*l*−*cis*−D, 50 μM) for 30 min. VAP, average path velocity; VSL, straight-line velocity; VCL, curvilinear velocity; ALH, amplitude of lateral head displacement; BCF, beat cross frequency. Bars indicate the mean ± SEM of three experiments. n = 500 for each sample. *$P < 0.05$.

Table S3. **Information about the primary antibodies used in immune detection**.

Antibody	Catalog Code	Source	Host	Dilution	
				IHC/IF	WB
NPPC	Sc374043	Santa Cruz Biotechnology	Rabbit	1:50	1:1000
p−SMAD3	9520	Cell Signaling Technology	Rabbit		1:1000
p−SMAD2/3	8828	Cell Signaling Technology	Rabbit		1:1000
SMAD2/3	8685	Cell Signaling Technology	Rabbit	1:100	1:1000
SMAD4	46535	Cell Signaling Technology	Rabbit	1:100	1:1000
TGFB1	ab92486	Abcam	Rabbit	1:200	1:1000
TGFBR1	ab31013	Abcam	Rabbit	1:200	1:1000
TGFBR2	ab186838	Abcam	Rabbit	1:200	1:1000
GAPDH	5174	Cell Signaling Technology	Rabbit		1:1000

IHC, Immunohistochemistry; IF, Immunofluorescence; WB, Western blotting.

Table S4. Primer sequences.

Gene	Primer
NPPA	F: ACCTGATGGATTTCAAGAATTTGCT<br>R: GGGGGCATAGCCTCATCTTC
NPPB	F: CAAGTCCTCCGGGGAATACG<br>R: ACCTCCTGAGCACATTGCAG
NPPC	F: AGGCAACAAGAAGGGTTTGTC<br>R: ACTAACATCCCAGGCCGCT
NPR2	F: CACCCACTTCATCAAGGCCAA<br>R: GATTCGGGGGTTCTCGGTAG
TGFB1	F: AGCCAGAGGCGGACTACTAC<br>R: AGGAATCATTGCTGTATTTCTGGT
TGFB2	F: TCCCCTCGGAAAATGCCATC<br>R: TCGCCTTCTGCTCTCGTTTT
TGFB3	F: CTGTGCGTGAATGGCTCTTG<br>R: CCTCACTGTCCACACCTTTGA
TGFBR1	F: GAGGCGACGGCATTCCAG<br>R: AGGCCAGATGGTGGCTTTC
TGFBR2	F: AACAGTGGGCAGGTGGGAAC<br>R: TTGGAACCAAAGGGTGGCTC
SMAD2	F: GTATGGACACAGGATCGCCA<br>R: TTGGACGGATCTGTGAAGCC
SMAD3	F: ACTACCAGAGGGTGGAGACC<br>R: AGAGGTTTGGAGAACCTGCG
SMAD4	F: TGTGGCTTCCACAAGTCAGC<br>R: AGGCTGGAATGCAAGCTCAT
GAPDH	F: AGCAATGCCTCCTGCACCACCA<br>R: TGAGTCCCTCCACGATGCCGAA
BMP15	F: AGGCCATTGGTTAATGGAGCA<br>F: GTGACATCCATCTCCGTCCA
GDF9	F: CCTCTACAACACTGTCCGGC<br>F: CACGATCCAGGTTAAACCGC

F, forward primer (5’-3’); R, reverse primer (5’-3’).
